# Post-Chemotherapy Foamy Histiocytes in Bone Marrow Aspiration of a Child with Acute Lymphoblastic Leukemia

**DOI:** 10.4274/tjh.galenos.2021.2020.0677

**Published:** 2021-02-25

**Authors:** Moeinadin Safavi, Zohreh Nozarian, Farzad Kompani

**Affiliations:** 1 Tehran, Iran; 2 Tehran University of Medical Sciences, Tehran, Iran; 3 Tehran University of Medical Sciences, Division of Hematology and Oncology, Children’s Medical Center, Pediatrics Center of Excellence, Tehran, Iran

**Keywords:** Acute lymphoblastic leukemia, Chemotherapy, Foamy macrophage

## To the Editor,

Foamy histiocytes are usual in a variety of disorders such as metabolic and/or lysosomal storage disorders as well as prolonged total parenteral nutrition [1,2]. Post-chemotherapy foamy histiocytes have been reported only twice in bone marrow aspiration (BMA) smears according to our investigation, in one case of acute myeloid leukemia and one case of metastatic adenocarcinoma of the prostate [3,4]. Here we report a new case of post-chemotherapy foamy histiocytes in BMA smears.

An 8-year-old girl presented with bone pain mostly in the left pelvis and lower limbs, fever, and weight loss for 2 weeks. Paleness and lymphadenopathy in the left submandibular and supraclavicular regions were found on physical examination. No other abnormal findings or hepatosplenomegaly were seen according to the general physical examination, abdominal sonography, or chest X-ray. Initial laboratory data showed a low hemoglobin level of 9.5 g/dL (reference range: 11-16 g/dL) and platelet count of 3,000/µL (reference range: 15,000-45,000/µL) with a leukocyte count of 5,500/µL (reference range: 4,000-10,000/µL). Other prominent laboratory findings were elevated erythrocyte sedimentation rate of 110 mm/h (reference range: 0-10 mm/h) and lactate dehydrogenase of 1,118 IU/L (reference range: 420-750 IU/L).

BMA was performed and showed more than 90% immature large cells with high nuclear to cytoplasm ratio and fine chromatin. In the flow cytometry of the BMA, these cells were positive for CD34, HLA-DR, TdT, CD10, CD19, and CD22 and were negative for CD3, CD20, CD117, and MPO. These findings confirmed the diagnosis of pre-B acute lymphoblastic leukemia NOS (pre-B ALL, NOS). Molecular analysis was negative for t(9;22)/BCR-ABL1, t(12;21)/ETV6-RUNX1, t(1;19)/TCF3-PBX1, and t(4;11)/KMT2A-AFF1.

Standard chemotherapy was started according to the Berlin-Frankfurt-Munster protocol including methylprednisolone at 60 mg/m^2^/day, intravenous vincristine at 1.5 mg/m^2^/day, intravenous daunomycin at 25 mg/m^2^/day, intramuscular L-asparaginase at 6,000 units/m^2^/day thrice weekly for 9 doses, and an intrathecal injection of cytosine arabinoside (Ara C; 30 mg), methotrexate (12.5 mg), and hydrocortisone (12.5 mg) [5].

**Figure 1:**
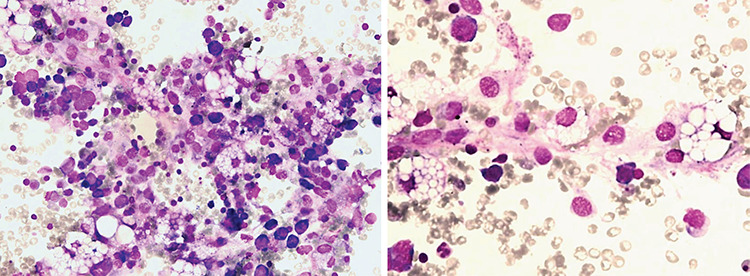
Smears showed multiple foamy histiocytes **(A, B)**.

BMA was performed 3 weeks later for evaluation of the early response to treatment after induction chemotherapy. Smears showed multiple foamy histiocytes (Figures 1A and 1B) and initial remission according to BMA cytology and immunophenotyping. In these histiocytes, there were many variably sized large vacuoles, which differed from sea-blue macrophages. No evidence of hemophagocytosis was observed. Unfortunately, metabolic screening had not been performed at birth for this patient. As she had no past medical history of any metabolic or lysosomal storage diseases, developmental delay, organomegaly, and/or metabolic crisis, there was no medical indication for evaluation of metabolic diseases. Interestingly, the foamy histiocytes disappeared in the subsequent BMA smears, which was taken as evidence against a metabolic disorder.

Post-chemotherapy foamy histiocytes were reported in only one previous case of acute myeloid leukemia 2 weeks after chemotherapy. That report suggested that the foamy histiocytes were related to degradation products of blasts phagocytized by the histiocytes. This finding might also be an idiosyncratic response to the chemotherapy [3]. In conclusion, a history of intensive chemotherapy should be considered in the list of differential diagnoses of foamy histiocytes in bone marrow specimens.
